# Unprotected Sex and Its Association with Other Risky Behaviours Among European Students: A Multinational Study

**DOI:** 10.3390/ijerph23010048

**Published:** 2025-12-30

**Authors:** Marco Scalese, Benedetta Ferrante, Sonia Cerrai, Sabrina Molinaro

**Affiliations:** 1 Institute of Clinical Physiology, National Research Council, 56124 Pisa, Italy; scalese@ifc.cnr.it (M.S.); benedettaferrante@cnr.it (B.F.); sonia.cerrai@cnr.it (S.C.); 2 Department of Epidemiology, Care and Public Health Research Institute (CAPHRI), Maastricht University, 6229 Maastricht, The Netherlands

**Keywords:** prevalence, sexual behaviour, ESPAD school survey, substance use, European countries

## Abstract

Background: Adolescents face unique challenges as they transition from childhood to adulthood, which can be marked by risky behaviours such as substance use and sexual activities. The present study analyses the relationship between risky sexual behaviour and the use of psychoactive substances, namely alcohol, cannabis, and other illegal substances, among students aged 15–16 years in 23 European countries, to investigate potential between-country differences. Method: Data were extracted from the 2019 European School Survey Project on Alcohol and Other Drugs (ESPAD) cross-sectional survey. Risky sexual behaviour was defined as self-reported unprotected sex. Substance use, other individual risk behaviours, and parenting indicators were investigated as key predictors. Results: A 9.8% of 16-year-old students in Europe reported sexual intercourse without a condom in the past year, and 7.8% had unprotected sex while not using alcohol/drugs, with a higher prevalence observed among males (8.5%) than among females (7.1%). Prevalence ranged from 3.2% in Georgia to 16.0% in Sweden. The multivariate analysis revealed significantly higher odds of engaging in sexual intercourse without a condom in illicit drug users (cannabis, inhalants, cocaine, ecstasy) and heavy episodic drinkers, students who went out in the evening, and those belonging to non-traditional families. Conclusions: Unprotected sex in the past year and substance use are strongly associated amid 16-year-old students in Europe. The prevalence of risky sexual behaviours across European countries does not follow a clear geographical pattern, suggesting that simple macro-level factors, such as broad regional or cultural groupings, may only partially explain prevalence differences.

## 1. Introduction

Adolescence is a critical developmental stage marked by significant physical, emotional, and cognitive changes. During this period, individuals are more likely to engage in exploratory behaviours, some of which can be risky and have long-term implications for their health and well-being [1]. Among these behaviours, sexual risk-taking and various forms of substance use/abuse are particularly concerning due to their potential to adversely affect adolescents’ physical and mental health [2].

Risky sexual behaviours in adolescents, such as early sexual initiation, unprotected sex, and multiple sexual partners, are associated with numerous negative outcomes including sexually transmitted infections (STIs), unintended pregnancies, and emotional distress [3,4,5]. Concurrently, the adolescence developmental stage also presents a heightened vulnerability to various forms of addiction, including substance abuse (alcohol, drugs), internet addiction, and gaming addiction. These addictive behaviours can exacerbate the propensity for sexual risk-taking by impairing judgement, increasing impulsivity, and diminishing the ability to make safe and informed decisions [6].

The interplay between risky sexual behaviours and addiction is complex and multifaceted. Research indicates that adolescents who engage in substance use are more likely to participate in unsafe sexual practices [7]. Similarly, internet and gaming addictions can lead to increased exposure to sexual content, potentially influencing sexual attitudes and behaviours [8]. Psychological factors such as sensation seeking, impulsivity, and a tendency towards thrill-seeking are common underpinnings that link both risky sexual behaviours and addictive tendencies in adolescents [9]. According to Owino and colleagues [10], individuals who have developed an anxious attachment, characterized by an intense need for relationships and simultaneously accompanied by a strong fear of being abandoned [11], are more likely to engage in risky sexual behaviours.

Moreover, social and environmental factors play a critical role in shaping these behaviours. Peer influence, family dynamics, socio-economic status, and exposure to media all contribute to the likelihood of an adolescent engaging in sexual risk-taking and developing addictive behaviours [12]. More specifically, peer group dynamics drive the adolescents’ behaviours, so it is the group identity and the behaviours that are enacted, influenced also by cultural and social values [13], that create a risk or a protective factor toward substance use or risky sexual behaviours [14,15,16,17]. Families also have an influence on adolescents’ sexual behaviours, especially in terms of parents’ styles [18], and for the imitation of parents’ own behaviours [19]. Socioeconomic status and social environment influence adolescents’ behaviours because of possible disparities that can occur, such as feelings of inequality and social injustice [20].

Families, schools, teachers, and peers play a vital role in fostering the healthy development of young people and supporting their transition to adulthood, particularly when they provide a supportive, safe, and positive environment [12]. Understanding the interrelationships among these factors is essential for developing effective prevention and intervention strategies.

The current study sought to investigate the prevalence of risky sexual behaviours among 16-year-old students in 23 countries across Europe and the overall relationship of unprotected sex and substance use (alcohol, cannabis, and other illegal substance), leisure time activities, and family structure and support. Data were drawn from the European School Survey Project on Alcohol and Other Drugs (ESPAD) [21], the largest cross-sectional survey that gathers information about legal and illegal substance use, as well as other sine substantia risky behaviours, among adolescent students in the European region.

## 2. Materials and Methods

### 2.1. Design and Selected Sample

Our analysis utilized data from the ESPAD study, a standardized cross-sectional survey initiated in 1995 to monitor comparable behavioural trends among 16-year-old students throughout Europe. Specifically, we focused on the 2019 collection wave, where 23 of the 35 participating countries collected optional information regarding unprotected sex, yielding a total sample size of 69,955 students included in the final analysis (see Table 1 for full list of included countries). The study methodology relies on representative national samples obtained through a random selection of schools and classes, ensuring the participation of the cohort of students born in 2003. The comprehensive, standardized ESPAD questionnaire gathers self-reported data on demographics, family environment, substance use, and engagement in several other behaviours (e.g., internet use, gaming, and gambling). To ensure cross-national comparability, the questionnaire was translated into the relevant national languages using a rigorous standard protocol, which included independent back-translation procedures. The ESPAD target population is precisely defined as students who reach the age of 16 years in the calendar year of the survey, are present in the classroom on the day of the survey, and are enrolled in regular, vocational, general, or academic studies. The sampling procedures demonstrated a high success rate: the average class participation rate was 85% (ranging from 21 to 100%), and the average students response rate reached 96% (ranging from 86 to 100%). Full details regarding the sampling design and individual country data are provided in Supplementary Table S1 and have been reported elsewhere [21].

### 2.2. Outcome Variable

Risky sexual behaviour was investigated through the following question: ‘During the LAST 12 MONTHS have you engaged in sexual intercourse without a condom?’ with the following response categories: ‘Never’, ‘Yes while using alcohol’, ‘Yes while using drugs’, ‘Yes, but NOT while using alcohol/drugs’. The prevalence of total unprotected sex and unprotected sex while not using alcohol/drugs are reported in Table 1. Unprotected sex while not using alcohol/drugs was the outcome variable for univariate and multivariate analyses.

### 2.3. Measures

Data concerning the family environment were captured through several measures, including family structure, which was assessed by determining who the student lives with (e.g., traditional family, single parents, stepfamily, or other living arrangements). We also evaluated the level of parental monitoring concerning Saturday night activities, students’ self-reported emotional support received from parents, and the presence of parental rule-setting defined both inside and outside the home. Individual behavioural characteristics were evaluated based on self-reported participation in various leisure time activities. This included the frequency of social outings (e.g., going around with friends for fun, going out in the evening), active participation in sports, engagement in reading for enjoyment, and pursuing other structured hobbies (e.g., playing an instrument, singing, drawing, writing). Finally, substance use was comprehensively measured by asking about engagement in heavy episodic drinking (HED) during the past year, alongside past-year use of cannabis, inhalants, amphetamines, methamphetamines, cocaine, crack, ecstasy, or heroin. Problematic use of cannabis in the last year was measured using the CAST (Cannabis Abuse Screening Test) questionnaire, a self-report screening questionnaire composed of 6 items scored based on a 5-point scale (0: “never”; 4: “very often”) [22,23]. It assesses problematic patterns of cannabis use by asking questions related to past-year cannabis consumption. The cut-off proposed by Bastiani et al. 2013 was used [23]. The cannabis use variable was categorized into three distinct levels: no use, non-problematic use, problematic use. A country-level indicator of the Socio-Demographic Index (SDI) was included as an adjusting variable to take into account the cross-country variability [24]. All the variables were coded dichotomously or in classes, and a detailed list of all the included variables is reported in Supplementary Table S2.

### 2.4. Statistical Analysis

Prevalence was initially computed (overall and by country) using standard percentages. The assessment of associations relied on multi-level mixed-effects logistic regression models, a necessary approach given the inherently hierarchical structure of the study data. The collected data exhibited a nested structure, with students’ characteristics (level 1) grouped within schools (level 2), which are, in turn, nested within countries (level 3). To mitigate potential bias arising from the correlation between student responses within the same geographical cluster, all statistical models incorporated random effects at both the school and country level. In this model, students represent the first level, schools form the second level, and countries make up the third level. The relationship between the predictor and outcome (slope) was assumed to be consistent across schools.

Initially, univariate analyses were conducted to evaluate the unadjusted relationship between each student characteristic and the outcome (reported as the Odds Ratio, OR). Subsequently, a multivariate multi-level mixed-effects logistic regression was performed, including all necessary variables, to determine the independent association of the potential predictors (reported as the adjusted Odds Ratio, aOR). A backward stepwise selection method was used to keep only the variables that maintained a significant association in the multivariate model. However, specific variables (SDI and sex) were retained as standard variables in the final model, irrespective of their statistical significance, due to their importance as established macro-level and demographic confounders. Statistical significance was defined as a *p*-value ≤ 0.05. All statistical analyses were executed using Stata software, version 13 (Stata Corporation, College Station, TX, USA).

### 2.5. Ethics Approval

The study was conducted across all ESPAD countries in strict adherence to the relevant European and national ethics rules. In accordance with the national laws of each participating country, the study protocol was approved or granted exemption by the appropriate institutional and/or national research ethics committee. A full description of the ethical aspects specific to each country is available in the ESPAD 2019 Methodology Report (Table 2) [25].

## 3. Results

Of the 69,955 students from 23 countries included in the analysis, 9.8% reported having unprotected sex globally, and 7.8% reported having sex without a condom (SWC) while not using alcohol/drugs in the past year. Overall, both behaviours showed higher prevalence among males (11.1% and 8.5%, respectively) than among females (8.7% and 7.1%, respectively), even though in 10 out of the 23 analyzed countries, females showed higher prevalence than males. Prevalence of SWC while not using alcohol/drugs ranged from 3.2% in Georgia to 16.0% in Sweden (Table 1).

**Figure 1:**
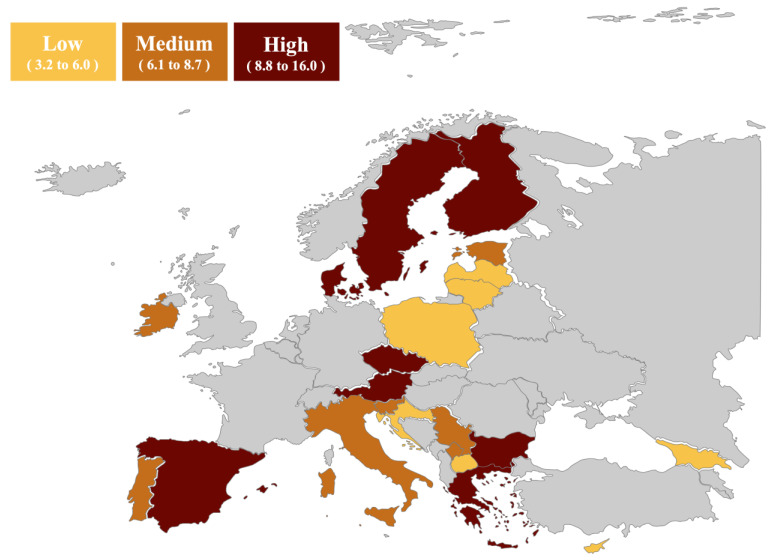
Percentage of students reporting sex without a condom while not using alcohol/drugs in the past year, stratified by tertiles.

We examined whether stratification by tertiles of SWC while not using alcohol/drugs in the past year identifies group of countries with different prevalence. Figure 1 shows no clear spatial distribution of risky sexual behaviours across Europe.

The descriptive analysis compares students who reported unprotected sexual intercourse while not using alcohol/drugs to all other students in the sample who did not report this specific high-risk behaviour. Univariate analyses highlighted marked differences in leisure activities and substance use between the groups. Students reporting SWC while not using alcohol/drugs were more likely to go out more often in the evening to discos, cafes, or parties (54.4% versus 36.0%). Conversely, they less frequently reported parental monitoring on Saturday night activities (82.7% vs. 90.9%), reading books for enjoyment (14.1% versus 21.3%), and having other hobbies (41.3% versus 47.6%). Compared to all other students, those reporting SWC while not using alcohol/drugs also showed a substantially higher prevalence of substance use history: for instance, alcohol intoxication in the last year (55.9% vs. 27.5%) and psychoactive substance use, including non-problematic cannabis use (22.8% vs. 8.9%), problematic cannabis use (8.9% vs. 1.9%), and ecstasy use (6.1% vs. 1.3%). Moreover, students who reported SWC while not using alcohol/drugs consistently showed a lower feeling of emotional support by family and a higher prevalence of belonging to a non-traditional family structure (Table 2).

The full results of the multivariate multi-level mixed-effects logistic regression are detailed in Table 2. The final model confirmed the independent association of several parameters with the outcome, with results presented as adjusted Odds Ratio (aOR) and their 95% confidence intervals.

The analysis revealed that even when isolating high-risk sexual behaviour that occurs while sober (i.e., SWC while not using alcohol/drugs), several individual factors remained significant independent predictors. While univariate analyses showed associations for all drug types, multivariate analyses only showed associations with unprotected sex for cannabis, ecstasy, inhalants, and cocaine. Adolescents who reported being intoxicated from drinking alcohol in the last year showed more than double the odds of engaging in SWC while not using alcohol/drugs (aOR = 2.20, *p* < 0.001). Similarly, the odds for those with non-problematic cannabis use (aOR = 2.18, *p* < 0.001) and problematic cannabis use (aOR = 2.69, *p* < 0.001) remained significant. Furthermore, using inhalants, ecstasy, and cocaine during the past year also showed a significant independent association with the outcome (aOR ranging from 1.24 to 1.51, *p* ≤ 0.003).

Engaging in certain leisure activities was also strongly associated with the outcome. Students who reported going out in the evening at least once a week had 57% increased odds (aOR = 1.57, *p* < 0.001), suggesting that social behaviours are major independent risk factors. Conversely, more internalized activities, such as reading books for enjoyment were significantly negatively associated (aOR = 0.76, *p* < 0.001).

Adolescents not living in a traditional family structure (single parents: aOR = 1.31; stepfamily: aOR = 1.58; other: aOR = 1.53, all *p* < 0.001) had significantly higher odds of the outcome. Higher parental monitoring remained a significant factor negatively associated with the outcome (aOR = 0.81, *p* < 0.001), as well as higher levels of family emotional support (aOR = 0.97, *p* < 0.001).

The analysis also found that other parameters significant in the univariate model lost their predictive power in the adjusted model. Specifically, high SDI and female sex were no longer significantly associated with the outcome in the fully adjusted model, suggesting that the influence of country development and gender is largely mediated by the individual risk factors and family context included in the model.

## 4. Discussion

Our findings reveal that 9.8% of adolescents in Europe reported condomless sexual intercourse, with 7.8% specifically engaging in unprotected sex while not using alcohol/drugs during the past year. Consistent with the established literature [26,27,28,29,30] we observed robust, significant associations between both drug use or HED in the past year and involvement in risky sexual behaviours.

The multivariate logistic regression analysis demonstrated that students who reported using substances such as inhalants, cocaine, ecstasy and cannabis had a higher likelihood of engaging in SWC while sober. This association may be rooted in the inherent characteristics of these psychoactive substances, which are known to impair cognitive functioning, alter rational decision-making ability, reduce inhibition, and generally increase risk-taking propensity [31]. Consequently, these changes predispose individuals to unsafe sexual practices, even in instances where the substance use and unprotected sex do not occur simultaneously.

We found that also having experienced HED in the past year was associated with SWC while not using alcohol/drugs, in line with other studies indicating an association of alcohol abuse with a higher number of sexual partners and a greater incidence of casual sex without protection [29,32]. However, relationship between HED and SWC should be studied more thoroughly. According to Scott-Sheldon et al. [33], alcohol consumption among heavy drinking college students leads to risky sexual behaviour, but the relation differs by gender and partner type. In general, college students who have experienced HED are more likely to engage in unprotected sex, even when sober, suggesting that this behaviour is a more general way of dealing with situations, less rational, and reflects a pattern of impulsivity, than those who do not have experienced HED [34,35].

The likelihood of unprotected sex also increases with casual partners that may ignore the perceived risk status, the main contextual factor influencing the decision to use a condom during sex [36].

In line with other studies, our findings have shown that higher levels of parental monitoring are associated with lower rates of risky sexual behaviours among adolescents [37,38]. This is especially true when parents maintain open communication about the risks of unprotected sex, such as the potential for sexually transmitted infections and unintended pregnancies [39]. Open communication about sex education can encourage critical thinking, with the possibility to analyze social norms and develop progressive attitudes toward gender and sexuality [40] and help with cultural complexity navigation and understanding taboos around sexuality [41]. Finally, it may contribute to mental well-being and healthier decision-making by helping students in exploring the emotional and psychological dimensions of sexuality. In the present work, we investigated parental monitoring defined as knowledge of students’ Saturday night activities and helping students make decisions. Our results underscored the importance of both dimensions in mitigating risky sexual behaviours among adolescents. Parents who actively engage in discussions about sexual health and maintain oversight of their children’s activities can significantly influence their decision-making processes regarding safe sex practices.

However, our findings also indicate that family structure itself represents a factor associated with risky sexual behaviour. Specifically, an increased risk was observed in association with non-traditional family structures. According to the institutionalization hypothesis [42,43], the normalization of non-traditional families and their gradual recognition as valid and functional family units, particularly expected with higher SDI levels, would also mitigate negative effects on adolescent behaviour, as these structures would be supported by cultural and institutional norms.

On the contrary, our results showed a direct association between non-intact family structures and unprotected sex, regardless of the country’s SDI. This suggests that the support provided by traditional families (in terms of supervision, stability, and protective norms) has a universally protective effect, which has not yet been fully replaced in non-traditional families, regardless of their cultural acceptance, as the association persists across countries with varying levels of socio-economic development, as measured by the SDI. Moreover, the absence of strong and stable institutions, such as traditional families, remains a cross-cutting vulnerability factor. It is also crucial to acknowledge that the effect of family structure on risky behaviours is rarely direct. Instead, it is often mediated by the adolescent’s peer group and social environment. Non-intact family structures may indirectly increase the adolescent’s vulnerability to SWC by leading to decreased parental supervision or increased exposure to peer groups that normalize risk-taking behaviours [43]. Furthermore, research suggests that the stress and transition associated with family changes can lead to higher impulsivity or externalizing behaviours, which are known predictors of sexual risk [43].

While the link between hobbies and condomless sex may not be straightforward, there are several social and psychological factors that can link these two domains. Hobbies can serve as a platform for self-exploration and expression, allowing teens to experiment with different aspects of their identities. For example, participation in creative activities can enhance self-esteem and provide a sense of belonging, which may influence their attitudes towards relationships and sex. Engaging in hobbies that promote positive peer interactions can lead to healthier social norms, including around sex. The type of hobbies adolescents pursue can also impact their social behaviours and attitudes towards risk-taking [44,45]. An important distinction among leisure activities resides in their structure. Structured activities, such as sports, physical activity, and creative activities, promote a range of skills and the development of positive and adaptive personal characteristics [46], to which can be added activities such as reading books, which allow one to come into contact with real and imaginary worlds and promote the empathic and abstract skills necessary for positive self-development [47,48]. The interplay of identity formation, peer relationships, and social behaviours highlights how hobbies can indirectly affect adolescents’ sexual choices.

Our findings have shown that actively participating in sports, athletics, or exercising is not associated with risky sexual behaviours among adolescents, indicating that various factors influence adolescent behaviour beyond just sports participation. Individual characteristics, familial influences, and peer dynamics all contribute to the decision-making processes regarding sexual activity [49].

The prevalence of sexual intercourse without a condom in Europe is influenced by a complex interaction of social, cultural, economic, educational, and health-related factors. The lack of a well-defined spatial distribution reflects the diverse influences of these factors. Consequently, there is no singular trend across Europe, and each country must be considered in the context of its unique sociocultural factors. Addressing the high rates of condomless sex in certain areas requires tailored interventions that take into account these diverse influences.

### 4.1. Strengths

The most significant strength of the study lies in the robust and consistent methodology used across a large number of European countries. To the best of our knowledge, this work on youth sexual behaviours comprises the largest geographic European area reported so far, resulting in a very large sample of adolescents and ensuring high data quality. The study reliability is furthermore supported by the use of homogeneous procedures for student recruiting and surveying across all countries, thereby guaranteeing maximum cross-sectional comparability of inclusion/exclusion criteria and outcome measures. This consistency is reinforced by the strict translation and back-translation process used to adapt the common questionnaire to the local language of each country, and by the high quality of data collection, reflected in the strong average students response rate (86%), and the national geographical coverage achieved by almost all samples, with minor exceptions noted in Supplementary Table S1.

### 4.2. Limitations

A significant limitation of the present study stems from the cross-sectional design of the ESPAD survey, which prevents the establishment of causal directionality. In addition, the findings are subject to common limitations of self-reporting (e.g., memory recall biases and social desirability biases), and restricted to the 16-year-old student population, thus limiting the generalizability to adolescents not involved in education or to those of different age groups.

The present study is also limited by the binary nature of the dependent variable, which recorded whether the students ever engaged in unprotected sexual intercourse during the past year, not accounting for the number of occasions. As the frequency of the behaviour was not assessed, the authors cannot ascertain the level of risk associated with repeated occurrences.

Another limitation concerns the design of the outcome variable: the question regarding unprotected sex while not using alcohol/drugs is derived from a pre-structured set of response categories within the ESPAD questionnaire. The combined nature of this question and its phrasing may introduce measurement bias, which may limit the generalizability of absolute prevalence estimates; however, the relative differences (Odds Ratios) are utilized for our comparative analysis of risk factors.

## 5. Conclusions

This study shed light on the complex interplay of factors that influenced condomless sex among European adolescents. Our findings confirmed prior research showing a strong association between substance use, such as inhalants, cocaine, ecstasy, cannabis, or heavy episodic drinking, and unprotected sex. Beyond substance use, our results emphasized the critical role of parental monitoring and family dynamics in shaping adolescent sexual behaviour. Higher levels of parental oversight, particularly through knowledge of adolescents’ activities and guidance in decision-making, were associated with reduced risky sexual behaviours. Interestingly, family structure emerged as a significant factor, with non-traditional family setups linked to an increased risk of unprotected sex, independent of a country’s SDI. Our study also highlighted the importance of structured hobbies in fostering social interactions and norms. Lastly, the lack of a clear spatial distribution of risky sexual behaviours across Europe underscores the need for localized interventions that addressed diverse sociocultural, economic, and health-related factors.

## Figures and Tables

**Table 1:** Percentage (%) of students reporting sex without a condom in the past year, stratified by country and sex.

	Unprotected Sex While Not Using Alcohol/Drugs			Total		
	Male	Female	Total	Male	Female	Total
Austria	9.9	10.1	10.0	13.0	13.1	13.1
Bulgaria	13.5	10.8	12.1	18.6	13.0	15.7
Croatia	5.3	3.0	4.2	8.4	3.7	6.2
Cyprus	7.1	3.4	5.0	12.3	4.9	8.1
Czechia	9.4	10.1	9.7	11.8	11.9	11.8
Denmark	11.5	13.3	12.4	16.5	16.9	16.7
Estonia	5.7	6.7	6.2	7.3	8.9	8.1
Faroes	4.1	8.2	6.2	4.9	9.8	7.4
Finland	7.8	9.6	8.7	10.3	12.1	11.2
Georgia	6.0	0.8	3.2	8.4	2.1	5.0
Greece	10.8	7.3	9.0	13.6	8.0	10.7
Ireland	8.3	4.7	6.4	12.4	7.9	10.1
Italy	9.3	7.7	8.5	11.8	8.8	10.4
Latvia	5.5	6.4	6.0	6.8	8.3	7.5
Lithuania	5.3	3.9	4.6	7.3	5.0	6.1
Poland	7.0	4.8	5.9	9.4	6.6	7.9
Portugal	7.0	8.5	7.8	8.0	9.2	8.7
Serbia	9.7	3.2	6.3	13.2	5.1	9.0
Slovenia	6.7	5.8	6.3	9.0	7.0	8.0
Spain	7.9	11.2	9.6	9.5	13.0	11.3
Sweden	15.0	17.1	16.0	16.6	18.3	17.5
North Macedonia	8.5	2.7	5.5	10.3	3.7	6.9
Kosovo	11.2	1.8	6.1	12.8	2.2	7.1
Total	8.5	7.1	7.8	11.1	8.7	9.8

**Table 2:** Multivariate multi-level logistic regression of main factors associated with unprotected sexual intercourse while not using alcohol/drugs. Adjusted Odds Ratio (aOR).

	aOR (95% CI)	*p*
Socio-Demographic Index (High)	1.06 (0.74–1.54)	0.731
Sex (female)	0.95 (0.88–1.01)	0.102
***Leisure time activities (at least once a week)***		
Read books for enjoyment (excluding schoolbooks)	0.76 (0.69–0.84)	<0.001
Go out in the evening (to a disco, cafe, party, etc.)	1.57 (1.46–1.68)	<0.001
Other hobbies (play an instrument, sing, draw, write)	0.92 (0.86–0.99)	0.021
***Family***		
Family structure (traditional family)	reference	
single parents	1.31 (1.19–1.42)	<0.001
stepfamily	1.58 (1.41–1.77)	<0.001
other	1.53 (1.33–1.76)	<0.001
Parents quite often/always know about Saturday night activities	0.81 (0.74–0.89)	<0.001
***Substance use***		
Intoxicated from drinking alcoholic beverages in the last year	2.20 (2.04–2.37)	<0.001
Cannabis use in the last year (No use)	reference	
Non problematic use	2.18 (2.00–2.39)	<0.001
Problematic use	2.69 (2.31–3.14)	<0.001
Inhalant use in the last year	1.24 (1.07–1.43)	0.003
Ecstasy use in the last year	1.51 (1.25–1.82)	<0.001
Cocaine use in the last year	1.47 (1.19–1.82)	<0.001
***Student’s feeling of emotional support*** *(Likert scale from 1-Very strongly disagree to 7-Very strongly agree)*		
My family is willing to help me make decisions	0.97 (0.95–0.98)	<0.001

aOR (95% CI): adjusted Odds Ratio and 95% confidence interval estimated from the final multivariate multi-level mixed-effects logistic regression.

## Data Availability

Data is unavailable due to the rules of the ESPAD consortium. However, the entire scientific community can have access to the data through an application process that can be found at this address: https://epid-prod.ifc.cnr.it/ESPAD-Appl (accessed on 15 September 2025).

## Supplementary Materials

The following supporting information can be downloaded at: https://www.mdpi.com/article/10.3390/ijerph23010048/s1, Table S1: Characteristics of the national samples by country. ESPAD, 2019; Table S2: Independent variables included in the model, questions, and codes. ESPAD, 2019; Table S3: Percentage (%) of students reporting sex without a condom in the past year (all categories), stratified by country; Table S4: Distribution of main behaviours adopted by students that reported sex without condom: not while using alcohol/drugs. Odds Ratio (OR) estimated separately on each variable (univariate).
