# Supplementary material for: Unprotected Sex and Its Association with Other Risky Behaviours Among European Students: A Multinational Study
Source: Int J Environ Res Public Health. 2025 Dec 30;23(1):48. doi: 10.3390/ijerph23010048 (PMC12841082; doi:10.3390/ijerph23010048)
Supplement: Supplementary file 1 [file ijerph-23-00048-s001.zip › ijerph-3906087-supplementary.pdf]

Supplementary Table S1. Characteristics of the national samples by country. ESPAD, 2019

Country	Class participation rate (%) (a)	Sample type	Students representativeness (%) (b)	Sex optional questions
Austria	92	Multistage stratified random	95	yes
Bulgaria	100	Multistage stratified random	95	yes
Croatia	94	Stratified random	98	yes
Cyprus	75	Multistage random	100	yes
Czechia	–	Multistage stratified random	> 95	yes
Denmark	21	Stratified random	100	yes
Estonia	80	Stratified random	100	yes
Faroes	100	Total	95	yes
Finland	79	Multistage stratified random	100	yes
France	100	Multistage stratified random	97	no
Georgia	51	Multistage random	100	yes
Germany	89	Systematic random	88	no
Greece	89	Stratified clustered random	92	yes
Hungary	74	Stratified random	99	no
Iceland	50	Total	96	no
Ireland	85	Stratified systematic random	98	yes
Italy	89	Multistage stratified random	99	yes
Kosovo	83	Multistage random	–	yes
Latvia	100	Stratified random	98	yes
Lithuania	99	Simple random	100	yes
Malta	99	Total	95	no
Monaco	100	Total	100	no
Montenegro	100	Total	94	no
Netherlands	–	Multistage random	98	no
North Macedonia	86	Systematic random	95	yes
Norway	58	Multistage stratified random	98	no
Poland	91	Stratified random	98	yes
Portugal	94	Stratified random	100	yes
Romania	–	Multistage random	90	no
Serbia	86	Multistage stratified random	86	yes
Slovakia	95	Stratified random	94	no
Slovenia	99	Stratified random	91	yes
Spain	90	Multistage stratified random	100	yes
Sweden	85	Multistage random	94	yes
Ukraine	96	Multistage stratified random	98	no
Average	**85**		**96**	

(a) Proportion of selected classes participating in the survey
(b) Proportion of ESPAD target students covered by the sampling frame
Note: data from ESPAD 2019 methodology
Not national geographical coverage: Finland, where the Åland Islands were not covered by the sampling frame. Cypro, only government-controlled areas were covered by the sampling frame. Georgia, the occupied territories of Abkhazia and South Ossetia were not covered by the sampling frame. Kosovo 4 % of the target population enrolled in schools in Northern Kosovo and/or functioning under the parallel structures of the Ministry of Education of Serbia within the other Serbian municipalities were not covered by the sampling frame

Supplementary Table S2. Independent variables included in the model, questions and codes. ESPAD, 2019

INDIPENDENT VARIABLES	QUESTIONS	CODES
Family environment:		
Family structure	- Which of the following people live in the same household with you?	Traditional family (Father and Mother) (0), Single parents (Father or Mother) (1), Stepfamily (Father with Stepmother or Mother with Stepfather) (2), Other (all the other combinations) (3)
Parents monitoring of Saturday night activities	- Does your mother or your father know where you spend Saturday nights?	Always/quite often (1) vs sometimes/usually don't' know (0)
Scholar's feeling of emotionally support by parents	- My family really tries to help me<br>- I get the emotional help and support I need from my family<br>- I can talk about my problems with my family<br>- My family is willing to help me make decisions	Scale from 1 (Very strongly disagree) to 7 (Very strongly agree)
Parents define rules at home and outside	- My parent(s) set definite rules about what I can do at home and/or outside	Seldom/almost never (0) vs almost always/often/sometimes (1)
Individual variables:		
Leisure time activities	How often do you:<br>- Actively participate in sports, athletics or exercising<br>- Read books for enjoyment (do not count schoolbooks)<br>- Go out in the evening (to a disco, cafe, party etc.)<br>- Other hobbies (play an instrument, sing, draw, write)	Never/a few times a year/1-2 a month (0) vs Almost every day/at least one a week (1)
Substance use:		
Substance use	- During the last 12 months, on how many occasions (if any) have you used:<br>- Heroin<br>- Cocaine<br>- Stimulants<br>- Inhalants<br>- Cannabis<br>- During the last 12 months, on how many occasions (if any) have you been intoxicated from drinking alcoholic beverages?	Never (0) vs Once or more (1)
Country-level indicator		
Socio-demographic Index (SDI)	composite indicator of development status strongly correlated with health outcomes. It is the geometric mean of 0 to 1 indices of total fertility rate under the age of 25, mean education for those ages 15 and older, and lag distributed income per capita	High vs other (Low, Low-middle, Middle, High-middle).

Supplementary Table S3. Percentage (%) of students reporting sex without a condom in the past year (all categories), stratified by country

	Unprotected sex in the past year			
	Never	Yes, while using alcohol	Yes, while using drugs	Yes, while not using alcohol/drugs
Austria	87,6	3,8	1,2	10,0
Bulgaria	84,5	3,1	1,1	12,1
Croatia	93,9	1,7	,4	4,2
Cyprus	91,9	1,5	1,7	5,0
Czechia	88,2	2,4	,4	9,7
Denmark	83,3	7,2	,8	12,4
Estonia	91,9	2,4	,5	6,2
Faroes	92,6	1,2	0,0	6,2
Finland	88,8	3,4	,5	8,7
Georgia	95,3	1,5	,7	3,2
Greece	90,0	1,5	,4	9,0
Ireland	89,9	3,4	,6	6,4
Italy	89,8	1,3	,8	8,5
Latvia	92,5	1,8	,1	6,0
Lithuania	93,9	1,6	,4	4,6
Poland	92,1	1,9	,4	5,9
Portugal	92,1	,8	,6	7,8
Serbia	91,3	1,9	,9	6,3
Slovenia	92,1	1,6	,4	6,3
Spain	89,0	1,6	,7	9,6
Sweden	82,7	2,7	,9	16,0
North Macedonia	94,1	1,9	1,8	5,5
Kosovo	92,9	,6	,3	6,1
**Total**	**90,4**	**2,2**	**,7**	**7,8**

Supplementary Table S4. Distribution of main behaviours adopted by students that reported sex without condom: not while using alcohol/drugs. Odds Ratio (OR) estimated separately on each variable (univariate).

	**Sex without condom last year**		**Univariate**	
	**No (%)**	**Yes (%)**	**OR (95% CI)**	***p***
Socio-demographic Index (High)	44.0	47.4	1.23 (0.87-1.73)	0.241
Sex (female)	51.9	47.0	0.85 (0.80-0.90)	<0.001
Leisure time activities (at least once a week)				
Actively participate in sports, athletics or exercising	80.5	80.3	0.91 (0.84-0.98)	0.013
Read books for enjoyment (excluding schoolbooks)	21.3	14.1	0.64 (0.59-0.70)	<0.001
Go out in the evening (to a disco, cafe, party etc.)	36.0	54.4	2.37 (2.23-2.53)	<0.001
Other hobbies (play an instrument, sing, draw, write)	47.6	41.3	0.84 (0.79-0.89)	<0.001
Family				
Family structure (traditional family)	73.8	63.5	reference	
single parents	15.0	18.7	1.48 (1.37-1.61)	<0.001
stepfamily	6.5	10.8	1.98 (1.79-2.19)	<0.001
other	4.7	7.1	1.83 (1.62-2.06)	<0.001
Parents define rules at home (almost always/often)	38.1	38.7	1.03 (0.97-1.10)	0.254
Parents define rules outside (almost always/often)	37.9	36.8	0.97 (0.91-1.03)	0.384
Parents know about Saturday night activities (almost always/often)	90.9	82.7	0.45 (0.41-0.49)	<0.001
Substance use				
Intoxicated from drinking alcoholic beverages in the last year	27.5	55.9	3.49 (3.28-3.71)	<0.001
Cannabis use in the last year (No use)	89.2	68.4	Reference	
Non problematic use	8.9	22.8	3.81 (3.52-4.13)	<0.001
Problematic use	1.9	8.9	6.65 (5.86-7.55)	<0.001
Inhalant use in the last year	3.5	7.5	2.30 (2.04-2.59)	<0.001
Ecstasy use in the last year	1.3	6.1	5.32 (4.62-6.14)	<0.001
Amphetamine use in the last year	0.9	4.1	4.74 (3.99-5.63)	<0.001
Methamphetamine use in the last year	0.7	3.1	4.95 (4.07-6.01)	<0.001
Cocaine use in the last year	1.1	4.8	5.04 (4.29-5.91)	<0.001
Crack use in the last year	0.7	2.8	4.35 (3.48-5.43)	<0.001
Heroin use in the last year	0.6	2.2	4.08 (3.23-5.15)	<0.001
Student's feeling of emotional support *(Likert scale from 1-Very strongly disagree to 7-Very strongly agree)*	**mean**	**mean**		
My family really tries to help me	6.1	5.8	0.92 (0.91-0.94)	<0.001
I get the emotional help and support I need from my family	5.7	5.4	0.92 (0.91-0.94)	<0.001
I can talk about my problems with my family	5.3	4.9	0.92 (0.91-0.93)	<0.001
My family is willing to help me make decisions	5.9	5.5	0.92 (0.90-0.93)	<0.001

OR (95% CI): Odds Ratio and 95% Confidence Interval estimated from univariate multi-level mixed-effects logistic regression
